# Accelerated nonstandard finite difference method for singularly perturbed Burger-Huxley equations

**DOI:** 10.1186/s13104-021-05858-4

**Published:** 2021-12-11

**Authors:** Masho Jima Kabeto, Gemechis File Duressa

**Affiliations:** Department of Mathematics, Jimma University, Jimma, Ethiopia

**Keywords:** Singularly perturbed Burger-Huxley equation, Accelerated nonstandard method, Accurate solution, Primary 65M06, 65M12, Secondary 65M15

## Abstract

**Objective:**

The main purpose of this paper is to present an accelerated nonstandard finite difference method for solving the singularly perturbed Burger-Huxley equation in order to produce more accurate solutions.

**Results:**

The quasilinearization technique is used to linearize the nonlinear term. A nonstandard methodology of Mickens procedure is used in the spatial direction and also within the first order temporal direction that construct the first-order finite difference approximation to solve the considered problem numerically. To accelerate the rate of convergence from first to second-order, the Richardson extrapolation technique is applied. Numerical experiments were conducted to support the theoretical results.

## Introduction

Singularly perturbed differential equations are typically characterized by a small perturbation parameter multiplied with the highest order derivative term. These types of equations appear in computational fluid dynamics, hydrodynamics, chemical reactor theory, financial modeling, mathematical biology [1–3]. The solutions of singularly perturbed problems exhibit layer. The conventional numerical methods on uniform meshes do not produce satisfactory numerical approximations for small values of the perturbation parameter. A uniformly convergent numerical method, which is a numerical method suitable for these problems and in which the error bound is independent of the size of the perturbation parameter (see [4, 5] and the references therein for more details).

In this work the singularly perturbed Burger-Huxley equations of the form:

1 $$\left\{ \begin{aligned} & L_{x,\varepsilon } u(x,t) \cong - \varepsilon \frac{{\partial^{2} u}}{{\partial x^{2} }} + \alpha u\frac{\partial u}{{\partial x}} + \frac{\partial u}{{\partial t}} - \beta (1 - u)(u - \gamma )u = 0,\,\,\,\,\,\forall (x,t) \in D, \\ \, & u(x,0) = u_{0} (x),\,\,\,\,x \in [0,1],\,\,\,\,\,u(0,t) = s_{0} (t),\,\,\,\,\,\,\,\,\,u(1,t) = s_{1} (t),\,\,\,\,\,\,\,t \in (0,T], \\ \end{aligned} \right.$$

where $0 < \varepsilon < < 1$ is a perturbation parameter. The solution domain $D = (0,1) \times (0,T]$, and $\alpha \ge 1$, $\beta \ge 0$, $\gamma \in (0,1)$ are given constants.

Burger-Huxley equation describes the interaction between convection, diffusion, and reaction processes that have numerous fascinating phenomena such as busting oscillation, population genetics, bifurcation, and so on [6–11]. A numerical method for Eq. (1) has been developed only in [6] using a robust adaptive grid method, a uniformly convergent method, and a singular perturbation approach respectively. However, all these methods considered the standard backward Euler scheme. To solve Eq. (1) the idea of using classical numerical methods is not an efficient approach to produce an accurate solution. This should be develop an accelerated nonstandard finite difference method. As a general rule, higher-order convergent methods are preferred as they provide well numerical approximations with low computational cost. So, in this paper, our objective is to propose the accelerated nonstandard finite difference method for solving Eq. (1) by applying the nonstandard procedures of Mickens [12] in the space direction. We provide an error analysis for the method and prove that it is uniformly convergent with second-order accuracy after applying the Richardson extrapolation technique. It is also shown that the method is computationally more efficient compared to some existing methods in the literature.

## Main text

### Numerical method

Consider equation under consideration in Eq. (1), re-written as:

2 $$\left\{ \begin{aligned} & \frac{\partial u}{{\partial t}} - \varepsilon \frac{{\partial^{2} u}}{{\partial x^{2} }} = F\left( {x,t,u,\frac{\partial u}{{\partial x}}} \right),\,\quad \forall (x,t) \in D, \\ & u(x,0) = u_{0} (x),\,\,\,\,x \in [0,1],\,\,\,\,u(0,t) = s_{0} (t),\,\,\,u(1,t) = s_{1} (t),\,\quad t \in (0,T], \\ \end{aligned} \right.$$

where $$F\left( {x,t,u,\frac{\partial u}{{\partial x}}} \right) = \beta (1 - u)(u - \gamma )u - \alpha u\frac{\partial u}{{\partial x}}$$.

Let us consider the homogenous part of Eq. (2):

3 $$\left\{ \begin{gathered} \frac{\partial u}{{\partial t}} - \varepsilon \frac{{\partial^{2} u}}{{\partial x^{2} }} = 0\,\quad \forall (x,t) \in D, \hfill \\ u(x,0) = u_{0} (x),\,\,\,\,x \in [0,1],\,\,\,\,u(0,t) = s_{0} (t),\,\,\,u(1,t) = s_{1} (t),\,\,\,t \in (0,T]. \hfill \\ \end{gathered} \right.$$

We look for a solution to the dimensionless heat equation of the form of Eq. (3), when $\varepsilon = 1$, using separation of variables, we get the solution to Eq. (3) as:

4 $$u(x,t) = u_{0} (x)\exp ( - \pi^{2} t),$$

that satisfies both the initial and boundary conditions given in Eq. (2). The formula in Eq. (4) will be used to guess intial approximation in the linearization process. Thus, to linearize Eq. (2), by applying the quasilinearization technique on the nonlinear term, for the reasonable initial guess of the form of Eq. (4) is given by:

5 $$u^{(0)} (x,t) = u_{0} (x)\exp ( - \pi^{2} t).$$

Thus, the nonlinear term $F\left( {x,t,u,\frac{\partial u}{{\partial x}}} \right)$ can be linearized initially as:

6 $$F\left( {x,t,u^{(1)} ,\frac{{\partial u^{(1)} }}{\partial x}} \right) \cong F\left( {x,t,u^{(0)} ,\frac{{\partial u^{(0)} }}{\partial x}} \right) + \left( {u^{(1)} - u^{(0)} } \right)\left. {\frac{\partial F}{{\partial u}}} \right|_{{u^{(0)} }} + \left( {\frac{{\partial u^{(1)} }}{\partial x} - \frac{{\partial u^{(0)} }}{\partial x}} \right)\left. {\frac{\partial F}{{\partial \left( {\frac{\partial u}{{\partial x}}} \right)}}} \right|_{{\frac{{\partial u^{(0)} }}{\partial x}}}.$$

Substituting Eq. (6) into Eq. (2) and inducing for iteration number $i$, we obtain the linearized differential equation.

7 $$\frac{{\partial u^{(i + 1)} }}{\partial t} - \varepsilon \frac{{\partial^{2} u^{(i + 1)} }}{{\partial x^{2} }} + a^{(i)} (x,t)\frac{{\partial u^{(i + 1)} }}{\partial x} + b^{(i)} (x,t)u^{(i + 1)} = f^{(i)} (x,t),$$

where $$\begin{aligned}a^{(i)} (x,t) &= - \left. {\,\,\frac{\partial F}{{\partial \left( {\frac{\partial u}{{\partial x}}} \right)}}} \right|_{{\left( {x,\,t,\,u^{(i)} ,\,\,\frac{{\partial u^{(i)} }}{\partial x}} \right)}} , \ldots b^{(i)} (x,t) \\ & \quad = - \left. {\,\frac{\partial F}{{\partial u}}} \right|_{{\,\left( {x,\,t,\,u^{(i)} ,\,\,\frac{{\partial u^{(i)} }}{\partial x}} \right)}} ,\end{aligned}$$ $$\begin{aligned}f^{(i)} (x,t) &= F\left( {x,t,u^{(i)} ,\frac{{\partial u^{(i)} }}{\partial x}} \right) \\ & \quad - \,\left. {\,u^{(i)} \frac{\partial F}{{\partial u}}} \right|_{{\left( {x,\,t,\,u^{(i)} ,\,\,\frac{{\partial u^{(i)} }}{\partial x}} \right)}} - \left. {\,\,\frac{{\partial u^{(i)} }}{\partial x}\frac{\partial F}{{\partial \left( {\frac{\partial u}{{\partial x}}} \right)}}} \right|_{{\left( {x,\,t,\,u^{(i)} ,\,\,\frac{{\partial u^{(i)} }}{\partial x}} \right)}}\end{aligned}$$.

Let *N* be a positive integer different from one, then discretize the interval $[0,\,T]$ on the temporal direction with uniform step length k. Hence, the interval $[0,\,T]$ is partitioned into *N* equal sub-intervals with each nodal point satisfies: $0 = t_{0} < t_{1} < \, \cdots \, < \,t_{N} = T.$ Thus, the temporal nodal points are generated by

8 $$t_{n} = nk,\,\,\quad \,k = \frac{T}{N},\,\quad \,n = 0,1,\, \ldots ,N.$$

Using Taylor’s series expansion, we have:

$$u^{n + 1} (x) = u^{n} (x) + k\frac{{\partial u^{n} (x)}}{\partial t} + \frac{{k^{2} }}{2}\frac{{\partial^{2} u^{n} (x)}}{{\partial t^{2} }} + \frac{{k^{3} }}{6}\frac{{\partial^{3} u^{n} (x)}}{{\partial t^{3} }} + \cdots$$, which gives:

9 $$\frac{{\partial u^{n} }}{\partial t} \cong \frac{{u^{n + 1} (x) - u^{n} (x)}}{k} + TE,$$

where the truncation term is $TE = - \frac{k}{2}\frac{{\partial^{2} u^{n} (x)}}{{\partial t^{2} }}$. This indicates, the error estimate:

10 $$\left\| E \right\|_{\infty } \le \,Ck,$$

for the arbitrary constant $C = \frac{1}{2}\left\| {\frac{{\partial^{2} u^{n} (x)}}{{\partial t^{2} }}} \right\|_{\infty }$. Substituting Eq. (9) into Eq. (7) gives the semi-discrete differential equation of the form:

11 $$\left\{ \begin{aligned} & \left( { - \varepsilon \frac{{\partial^{2} u^{(i + 1)} }}{{\partial x^{2} }} + a^{(i)} \frac{{\partial u^{(i + 1)} }}{\partial x} + \left( {b^{(i)} + \frac{1}{k}} \right)u^{(i + 1)} } \right)(x,t_{n + 1} ) = \left( {f^{(i)} + \frac{1}{k}u^{(i)} } \right)(x,t_{n + 1} ), \\ \, & \,u(x,0) = u_{0} (x),\,\,\,\,\,x \in [0,\,1],\,\,\,\,u(0,t_{n + 1} ) = s_{0} (t_{n + 1} ),\,\,\,\,\,\,\,\,u(1,t_{n + 1} ) = s_{1} (t_{n + 1} ). \\ \end{aligned} \right.$$

Let us consider the singularly perturbed convection–diffusion differential equation of the form:

12 $$\varepsilon \frac{{d^{2} u(x)}}{{dx^{2} }} + \eta \frac{du(x)}{{dx}} - \theta u(x) = 0,\,\,\,x \in (0,\,1),$$

where $\eta > 0$ and $\theta \ge 0$ are constants. Then, Eq. (12) has two linearly independent solutions as:

13 $$u_{1} (x) = \exp (\lambda_{1} x),\,\quad {\text{and}}\quad \,u_{2} (x) = \exp (\lambda_{2} x),$$

for the constants $\lambda_{1} = \frac{{ - \eta + \sqrt {\eta^{2} + 4\varepsilon \theta } }}{2\varepsilon },\quad \,{\text{and}}\quad \lambda_{2} = \frac{{ - \eta - \sqrt {\eta^{2} + 4\varepsilon \theta } }}{2\varepsilon }$. Let *M* be is a positive integer, and the interval $[0,\,1]$ on the spatial direction be divided into *M* equal sub-intervals through the nodes $x_{m} = mh,\quad m = \frac{1}{M},\,\quad \,m = 0,\,1,\, \ldots ,M$, and the approximate solution to $u(x)$ at the grid points $x_{m} ^{\prime}s$ is denoted by $u_{m} ^{\prime}s$. The theory of difference (see [12]), shows that the second-order linear difference equation:

14 $$\begin{aligned} & u_{m - 1} \left( {\exp (\lambda_{2} h) - \exp (\lambda_{1} h)} \right) - u_{m} \left( {\exp (\lambda_{2} - \lambda_{1} )h - \exp (\lambda_{1} - \lambda_{2} )h} \right) \\ & \quad + u_{m + 1} \left( {\exp ( - \lambda_{1} h) - \exp (\lambda_{2} h)} \right) = 0. \\ \end{aligned}$$

Substituting the values of $\lambda_{1}$ and $\lambda_{2}$ into Eq. (13), we get:

15 $$u_{m - 1} \exp \left( {\frac{ - \eta h}{{2\varepsilon }}} \right) - 2\cosh \left( {\frac{{h\sqrt {\eta^{2} + 4\varepsilon \theta } }}{2\varepsilon }} \right) + u_{m + 1} \exp \left( {\frac{\eta h}{{2\varepsilon }}} \right) = 0.$$

This Eq. (15) is the exact difference scheme of Eq. (12), in the wisdom that the difference Eq. (15) has the general solution $u_{m} = C_{1} \exp (\lambda_{1} x_{m} ) + C_{2} \exp (\lambda_{2} x_{m} )$ as the differential Eq. (12). Since, $\theta \ge 0$, we require the exact scheme for the reduced case. To this end, the exact scheme corresponding to $\varepsilon \frac{{d^{2} u(x)}}{{dx^{2} }} + \eta \frac{du(x)}{{dx}} = 0.\,\,$ Thus, Eq. (15) is deduced to:

16 $$u_{m - 1} \exp \left( {\frac{ - \eta h}{{2\varepsilon }}} \right) - 2\cosh \left( {\frac{\eta h}{{2\varepsilon }}} \right)u_{m} + u_{m + 1} \exp \left( {\frac{\eta h}{{2\varepsilon }}} \right) = 0.$$

Multiplying both sides of Eq. (16) by $\exp \left( {\frac{\eta h}{{2\varepsilon }}} \right)$, and incorporating the term $u_{m + 1} - u_{m}$ into this equation, we get:

17 $$u_{m - 1} - 2u_{m} + u_{m + 1} + (u_{m + 1} - u_{m} )\left( {\exp \left( {\frac{\eta h}{\varepsilon }} \right) - 1} \right) = 0.$$

Consequently, Eq. (17) can be transformed into:

18 $$\varepsilon \frac{{u_{m - 1} - 2u_{m} + u_{m + 1} }}{{\phi^{2} }} + \eta \frac{{u_{m + 1} - u_{m} }}{h} = 0,$$

where $$\phi^{2} = \frac{h\varepsilon }{\eta }\left( {\exp (\frac{\eta h}{\varepsilon }) - 1} \right)$$.

When we come to the Burger-Huxley equation, the differential equation under consideration the full discretization of $\overline{D}_{M}^{N}$, denoting to approximation $u(x_{m} ,t_{n} )$ by $U_{m}^{n}$. Then, the nonstandard finite difference rules developed for ODE above can be extended for PDE is given by:

19 $$\left\{ \begin{aligned} L_{M}^{N} U \cong & - \varepsilon \left( {\frac{{U_{m - 1}^{n + 1} - 2U_{m}^{n + 1} + U_{m + 1}^{n + 1} }}{{\phi_{m}^{2} }}} \right)^{i + 1} + \left( {a_{m}^{n + 1} } \right)^{i + 1} \left( {\frac{{U_{m}^{n + 1} - U_{m - 1}^{n + 1} }}{h}} \right)^{i + 1} \\ & \quad + \left( {\left( {b_{m}^{n + 1} } \right)^{i + 1} + \frac{1}{k}} \right)\left( {U_{m}^{n + 1} } \right)^{i + 1} = \left( {f_{m}^{n + 1} } \right)^{i + 1} + \frac{1}{k}\left( {U_{m}^{n} } \right)^{i + 1} , \\ U_{m}^{0} = & u_{0} (x_{m} ),\,\,\,\,\,\,U_{0}^{n + 1} = s_{0} (t_{n + 1} ),\,\,\,\,\,\,\,\,U_{M}^{n + 1} = s_{1} (t_{n + 1} ), \\ \end{aligned} \right.$$

where $i = 0,1,\,\,...$ iteration number, and $\phi_{m}^{2} = \frac{h\varepsilon }{{a_{m}^{n + 1} }}\left( {\exp \left( {\frac{{ha_{m}^{n + 1} }}{\varepsilon }} \right) - 1} \right)$.

## Stability of the scheme

A partial differential equation is well-posed if the solution of the partial differential equation is exists, and depends continuously on the initial condition and boundary conditions. The Von Neumann stability technique is applied to investigate the stability of the developed scheme in Eq. (19), by assuming that the solution of Eq. (19) at the grid point $\left( {x_{m} ,t_{n} } \right)$ is given by:

20 $$U_{m}^{n} = \mathop \xi \nolimits^{n} \mathop e\nolimits^{im\theta }.$$

where $i = \sqrt { - 1} ,\,\,\,\theta$ is the real number and $\xi$ is the amplitude factor.

Now, considering at the first iteration and putting Eq. (20) into the homogeneous scheme part of Eq. (19) gives:

$$\begin{aligned} & \frac{ - \varepsilon }{{\phi_{m}^{2} }}\left( {\mathop \xi \nolimits^{n + 1} \mathop e\nolimits^{{i\left( {m - 1} \right)\theta }} - 2\mathop \xi \nolimits^{n + 1} \mathop e\nolimits^{im\theta } + \mathop \xi \nolimits^{n + 1} \mathop e\nolimits^{i(m + 1)\theta } } \right) + \frac{{a_{m}^{n + 1} }}{h}\left( {\mathop \xi \nolimits^{n + 1} \mathop e\nolimits^{im\theta } - \mathop \xi \nolimits^{n + 1} \mathop e\nolimits^{i(m - 1)\theta } } \right) \\ & \quad + b_{m}^{n + 1} \mathop \xi \nolimits^{n + 1} \mathop e\nolimits^{im\theta } + \frac{1}{k}\left( {\mathop \xi \nolimits^{n + 1} \mathop e\nolimits^{im\theta } - \mathop \xi \nolimits^{n} \mathop e\nolimits^{im\theta } } \right) = 0. \\ \end{aligned}$$

Then, solving for the amplitude factor $\xi$, yields:

$$\begin{aligned} \xi = & \frac{\frac{1}{k}}{{\frac{ - \varepsilon }{{\phi_{m}^{2} }}\left( {\mathop e\nolimits^{ - i\theta } - 2 + \mathop e\nolimits^{i\theta } } \right) + \frac{{a_{m}^{n + 1} }}{h}\left( {1 - \mathop e\nolimits^{ - i\theta } } \right) + b_{m}^{n + 1} + \frac{1}{k}}} \\ \,\,\,\,\, = & \frac{1}{{1 - \frac{ - \varepsilon k}{{\phi_{m}^{2} }}\left( {\mathop e\nolimits^{ - i\theta } - 2 + \mathop e\nolimits^{i\theta } } \right) + \frac{{ka_{m}^{n + 1} }}{h}\left( {1 - \mathop e\nolimits^{ - i\theta } } \right) + kb_{m}^{n + 1} }}. \\ \end{aligned}$$

The condition of stability is $\xi \,\,\, \le 1$ and for sufficiently small *k*, we have $\xi \,\,\, = 1$. Hence, the scheme given in Eq. (19) is stable. Thus, the scheme in Eq. (19) is unconditionally stable.

## Consistency of the scheme

The local truncation error $T(h,k)$ between the operator on the exact solution $u_{m}^{n}$ to Eq. (7) and the approximate solution $U_{m}^{n}$ to Eq. (19) at the fixed $i = 0$ iteration is given by:

21 $$\begin{aligned} T(h,k) = & \frac{{\partial u_{m}^{n + 1} }}{\partial t} - \varepsilon \frac{{\partial^{2} u_{m}^{n + 1} }}{{\partial x^{2} }} + a_{m}^{n + 1} \frac{{\partial u_{m}^{n + 1} }}{\partial x} + b_{m}^{n + 1} u_{m}^{n + 1} \\ \,\,\,\,\,\,\,\,\,\,\,\,\, & \quad \, - \left( {\frac{{U_{m}^{n + 1} - U_{m}^{n} }}{k} - \varepsilon \frac{{U_{m + 1}^{n + 1} - 2U_{m}^{n + 1} + U_{m - 1}^{n + 1} }}{{\phi_{m}^{2} }} +\, a_{m}^{n + 1} \frac{{U_{m}^{n + 1} - U_{m - 1}^{n + 1} }}{h} + b_{m}^{n + 1} U_{m}^{n + 1} } \right). \\ \end{aligned}$$

Using Taylor’s series expansion, we have:

22 $$\frac{{U_{m}^{n + 1} - U_{m}^{n} }}{k} = \frac{{\partial u_{m}^{n + 1} }}{\partial t} + \frac{k}{2}\frac{{\partial^{2} u_{m}^{n + 1} }}{{\partial t^{2} }} + O(k^{2} ).$$

23 $$U_{m + 1}^{n + 1} - 2U_{m}^{n + 1} + U_{m - 1}^{n + 1} = h^{2} \frac{{\partial^{2} u_{m}^{n + 1} }}{{\partial x^{2} }} + O(h^{4} ).$$

24 $$\frac{{U_{m}^{n + 1} - U_{m - 1}^{n + 1} }}{h} = \frac{{\partial u_{m}^{n + 1} }}{\partial x} + \frac{h}{2}\frac{{\partial^{2} u_{m}^{n + 1} }}{{\partial x^{2} }} + O(h^{2} ).$$

Substituting Eqs. (22)–(24) into Eq. (21), which implies:

25 $$T(h,k) = - \frac{k}{2}\frac{{\partial^{2} u_{m}^{n + 1} }}{{\partial t^{2} }} - \frac{h}{2}a_{m}^{n + 1} \frac{{\partial^{2} u_{m}^{n + 1} }}{{\partial x^{2} }} - \left( {1 - \frac{{h^{2} }}{{\phi_{m}^{2} }}} \right)\varepsilon \frac{{\partial^{2} u_{m}^{n + 1} }}{{\partial x^{2} }} + O(h^{2} + k^{2} ).$$

Now, the values of $\phi_{m}^{2}$ provided in Eq. (19) can be expanded as:

$$\phi_{m}^{2} = h^{2} + \frac{{h^{3} }}{2!}\frac{{a_{m}^{n + 1} }}{\varepsilon } + \frac{{h^{4} }}{3!}\left( {\frac{{a_{m}^{n + 1} }}{\varepsilon }} \right)^{2} + \frac{{h^{5} }}{4!}\left( {\frac{{a_{m}^{n + 1} }}{\varepsilon }} \right)^{3} + \cdots.$$

Thus, the truncation error is

26 $$T(h,k) = - \frac{k}{2}\frac{{\partial^{2} u_{m}^{n + 1} }}{{\partial t^{2} }} - \frac{h}{2}a_{m}^{n + 1} \frac{{\partial^{2} u_{m}^{n + 1} }}{{\partial x^{2} }} + O(h^{2} + k^{2} ).$$

Therefore, the norm of the local truncation error can be written as:

27 $$\left| {T(h,k)} \right| = \left| {L\left( {u_{m}^{n + 1} - U_{m}^{n + 1} } \right)} \right| \le C_{1} h + C_{2} k,$$

where $$C_{1} = \frac{1}{2}\left\| {\frac{{\partial^{2} u_{m}^{n + 1} }}{{\partial t^{2} }}} \right\|_{\infty } ,\,\,\,\,{\text{and}}\,\,C_{2} = \frac{1}{2}\left\| {a_{m}^{n + 1} \frac{{\partial^{2} u_{m}^{n + 1} }}{{\partial x^{2} }}} \right\|_{\infty } .$$

To accelerate the rate of convergence, we apply the Richardson extrapolation techniques on Eq. (27). Assume that $U(h,k)$ denote the approximate value of $u(x_{m} ,t_{n} )$ with the mesh length of *h* and *k*. The approximate solution $U\left( {\frac{h}{2},\frac{k}{2}} \right)$ also denotes the value of $u(x_{m} ,t_{n} )$ obtained by using the same method with step length $\frac{h}{2}$ and $\frac{k}{2}$, then the order of convergence for the two approximate solutions can be written as:

28 $$\left\{ \begin{aligned} & u(x_{m} ,t_{n} ) - U(h,k) \equiv C(h + k) + O(h^{2} + k^{2} ), \\ & u(x_{m} ,t_{n} ) - U\left( {\frac{h}{2},\frac{k}{2}} \right) \equiv C\left( {\frac{h}{2} + \frac{k}{2}} \right) + O(h^{2} + k^{2} ), \\ \end{aligned} \right.$$

where *C* is a constant independent of the perturbation and mesh parameters.

Eliminating C from Eq. (28), gives:

29 $$u(x_{m} ,t_{n} ) - 2U\left( {\frac{h}{2},\frac{k}{2}} \right) + U(h,k) \equiv O(h^{2} + k^{2} ).$$

Let us denote the combination of the two approximate solutions in Eq. (29) by:

$$U_{(h,k)}^{ext} = 2U\left( {\frac{h}{2},\frac{k}{2}} \right) + U(h,k).$$

Then, it is re-written as:

30 $$u(x_{m} ,t_{n} ) - U_{(h,k)}^{ext} = O(h^{2} + k^{2} ).$$

This indicates the method is accelerated to second-order. Hence, we have

31 $$\left| {u(x_{m} ,t_{n} ) - U_{(h,k)}^{ext} } \right| \le C(h^{2} + k^{2} ).$$

Therefore, the proposed method is second-order uniformly convergent. Thus, the right hand side hand of Eq. (27) vanishes as $k \to 0\,\,{\text{and}}\,\,h \to 0$ implies $T(h,k) \to 0$. Hence, the scheme is consistent with the order of convergence $O\left( {\mathop k\nolimits^{2} + \mathop h\nolimits^{2} } \right)$. Ended, the scheme developed in Eq. (19), is convergent. A consistent and stable finite difference method is convergent by Lax's equivalence theorem [2]. For further convergence analysis, one can see the works provided in [5, 11, 13, 14].

## Numerical illustration and discussions

The exact solution for the considered examples is not available. Hence, the maximum absolute errors are calculated by the double mesh principle, [6], for before and after applying the Richardson extrapolation technique respectively by:

$$E_{M}^{N} = \mathop {\max }\limits_{{\forall (x_{m} ,t_{n} ) \in \overline{D}}} \left| {U_{m}^{n} - U_{2m}^{2n} } \right|,\quad {\text{and}}\quad \left( {E_{M}^{N} } \right)^{ext} = \mathop {\max }\limits_{{\forall (x_{m} ,t_{n} ) \in \overline{D}}} \left| {\left( {U_{m}^{n} } \right)^{ext} - \left( {U_{2m}^{2n} } \right)^{ext} } \right|,$$

where $U_{m}^{n}$ and $U_{2m}^{2n}$ are approximate solutions evaluated on $\overline{D}_{M}^{N}$ and $\overline{D}_{2M}^{2N}$ respectively. Similarly, its extrapolated are induced. The corresponding rate of convergences is determined by:

$$R_{M}^{N} = \frac{{\log (E_{M}^{N} ) - \log (E_{2M}^{2N} )}}{\log (2)},\quad {\text{and}}\quad \left( {R_{M}^{N} } \right)^{ext} = \frac{{\log \left( {\left( {E_{M}^{N} } \right)^{etx} } \right) - \log \left( {\left( {E_{2M}^{2N} } \right)^{ext} } \right)}}{\log (2)}.$$

### ***Example 1.***

Consider the singularly perturbed Burgers-Huxley equation:

$$\left\{ \begin{gathered} \left( {\frac{\partial u}{{\partial t}} - \varepsilon \frac{{\partial^{2} u}}{{\partial x^{2} }} + u\frac{\partial u}{{\partial x}} - (1 - u)(u - 0.5)u} \right)(x,t) = 0,\,\,\,\,\,(x,t) \in (0,1) \times (0,\,1], \hfill \\ u(x,0) = x(1 - x^{2} ),\,\,\,\,\,\,0 < x < 1,\,\,\,\,\,\,\,u(0,t) = u(1,t) = 0,\,\,\,\,\,\,t \in [0,1]. \hfill \\ \end{gathered} \right.$$

### ***Example 2.***

Consider the following singularly perturbed Burgers’ equation:

$$\left\{ \begin{gathered} \left( {\frac{\partial u}{{\partial t}} - \varepsilon \frac{{\partial^{2} u}}{{\partial x^{2} }} + u\frac{\partial u}{{\partial x}}} \right)(x,t) = 0,\,\,\,\,\,(x,t) \in (0,1) \times (0,\,1], \hfill \\ u(x,0) = x(1 - x^{2} ),\,\,\,\,\,\,0 < x < 1,\,\,\,\,\,u(0,t) = u(1,t) = 0,\,\,\,\,\,\,t \in [0,1]. \hfill \\ \end{gathered} \right.$$

Tables 1 and 2 show the maximum absolute errors that demonstrate the validity of the present method and errors are monotonically decreasing behavior with increasing the number of intervals which confirm the convergence of the method. Table 3 validate that the corresponding rate of convergence. Thus, the proposed method is second-order convergent. Furthermore, the method gives a more accurate solution than some existing methods in the literature.

**Table 1:** Comparison of maximum absolute errors for Example 1

$\varepsilon \downarrow \,M/N \to$	32/20	64/40	128/80	256/160	512/320
*After extrapolation*					
$2^{ - 6}$	1.1939e−04	3.4613e−05	9.3412e−06	4.2951e−06	2.3138e−06
$2^{ - 8}$	1.7054e−04	5.6853e−05	1.5929e−05	4.2117e−06	1.0822e−06
$2^{ - 10}$	2.1124e−04	1.0056e−04	3.8345e−05	1.1640e−05	3.1477e−06
$2^{ - 12}$	2.0194e−04	7.9291e−05	5.5162e−05	3.0062e−05	1.0964e−05
$2^{ - 18}$	2.7271e−04	7*.*2971e−05	1.8774e−05	4*.*6058e−06	1.8644e−06
*Before extrapolation*					
$2^{ - 6}$	3.3878e−03	1.7453e−03	8*.*8211e−04	4.4321e−04	2.2212e−04
$2^{ - 8}$	4.0171e−03	2.0163e−03	1.0020e−03	4.9855e−04	2.4852e−04
$2^{ - 10}$	4*.*4610e−03	2.2241e−03	1.0800e−03	5.2600e−04	2.5871e−04
$2^{ - 12}$	4*.*6996e−03	2*.*4258e−03	1.2014e−03	5.7660e−04	2.7486e−04
$2^{ - 18}$	4*.*7584e−03	2*.*5003e−03	1.2810e−03	6.4852e−04	3.2604e−04
Res*ults in *[6]					
$2^{ - 6}$	2.5289e−02	1.7672e−02	9.0066e−03	4.8378e−03	2.5035e−03
$2^{ - 8}$	3.8607e−02	1.9497e−02	1.1221e−02	6.2852e−03	3.3405e−03
$2^{ - 10}$	9.3183e−02	7.0120e−02	4.4773e−02	2.0546e−02	1.0545e−02
$2^{ - 12}$	1.7017e−01	1.0083e−01	6.2216e−02	3.9526e−02	2.0493e−02
$2^{ - 18}$	2.5614e−01	2.1031e−01	1.3406e−01	8.5618e−02	4.8834e−02

**Table 2:** Comparison of maximum absolute errors for Example 2

$\varepsilon \downarrow \,M/N \to$	32/20	64/40	128/80	256/160	512/320
*After extrapolation*					
$2^{ - 6}$	1*.*0956e−04	2.8034e−05	7.6248e−06	4.1959e−06	2.2851e−06
$2^{ - 8}$	1.5301e−04	4.9218e−05	1.3827e−05	3.6543e−06	9.3878e−07
$2^{ - 10}$	2.0315e−04	9.4930e−05	3.6697e−05	1.1291e−05	3.0711e−06
$2^{ - 12}$	1.9354e−04	8.7202e−05	5.8753e−05	3.1369e−05	1.1375e−05
$2^{ - 18}$	2.7286e−04	7.2496e−05	1.8396e−05	4.4949e−06	2.6628e−06
*Results in* [6]					
$2^{ - 6}$	3.8767e−02	1.8983e−02	9.6122e−03	5.0867e−03	2.6211e−03
$2^{ - 8}$	4.4450e−02	2.0109e−02	1.0519e−02	5.9653e−02	3.1896e−03
$2^{ - 10}$	8.3339e−02	6.6120e−02	4.0769e−02	1.9284e−02	9.1775e−03
$2^{ - 12}$	1.8762e−01	8.4106e−02	5.7234e−02	3.309e−02	1.9041e−02
$2^{ - 18}$	2.8299e−01	1.7036e−01	1.1805e−01	7.4251e−02	4.1879e−02

**Table 3:** Comparison of rate of convergence for Example 1

$\varepsilon \downarrow \,M/N \to$	32/20	64/40	128/80	256/160
*After extrapolation*				
$2^{ - 6}$	1*.*9098	1.9894	1.7283	1*.*6506
$2^{ - 8}$	1.8727	1.9458	1.9784	1.9936
$2^{ - 10}$	1.8710	1.9392	1.9706	1.9868
$2^{ - 12}$	1.8715	1.9350	1.9696	1.9852
$2^{ - 18}$	1.8713	1.9339	1.9692	1.9845
*Before extrapolation*				
$2^{ - 6}$	0.8839	0.9566	0.9989	1.0228
$2^{ - 8}$	0.8839	0.9566	0.9989	1.0228
$2^{ - 10}$	0.8769	0.9385	0.9695	0.9851
$2^{ - 12}$	0.8778	0.9388	0.9686	0.9846
$2^{ - 18}$	0.8782	0.9390	0.9679	0.9845
*Results in* [6]				
$2^{ - 6}$	0.5170	0.9724	0.8966	0.9504
$2^{ - 8}$	0.9856	0.7971	0.8362	0.9119
$2^{ - 10}$	0.4102	0.6472	1.1238	0.9623
$2^{ - 12}$	0.7550	0.6966	0.6544	0.9477
$2^{ - 18}$	0.2844	0*.*6496	0.6469	0.8100

## Conclusion

The accelerated nonstandard finite difference method is presented for solving the singularly perturbed Burger-Huxley equation. The nonlinear terms are linearized by the quasilinearization technique. First-order finite difference approximation for the discretization of a time derivative and the nonstandard methodology of Micken’s procedure is applied for the spatial derivatives. To accelerate the convergence of the method, the Richardson extrapolation technique is applied. It is provided that from numerical results, the method gives a better accurate solution with a higher order of convergence than some existing methods in the literature. Therefore, the presented method is second-order convergent and gives an accurate solution for solving the singularly perturbed Burger-Huxley equation.

## Limitations

During the quasi-linearization process we used only the first iteration. If more number of iterations were done, the proposed scheme can have more accurate solution than the existing results. Further, the scheme can more illustrate the physical behaviour of the problem under consideration.

## Data Availability

No additional data is used for this research work.

## Abbreviations

ODE: Ordinary differential equation
TE: Truncation errors
